# Prospective Evaluation of Fetal Hemoglobin Expression in Maternal Erythrocytes: An Analysis of a Cohort of 345 Parturients

**DOI:** 10.3390/diagnostics13111873

**Published:** 2023-05-27

**Authors:** Laurence Blain, Christian Watier, Xiaoduan Weng, Andre Masse, Marie-Josée Bédard, Nazila Bettache, Florence Weber, Michele Mahone, Stéphanie Forté, Vincent-Philippe Lavallée, Pierre-Olivier Gaudreau, Michael J. Newmarch, Denis Soulières

**Affiliations:** 1 Department of Medicine, Centre Hospitalier de l’Université de Montréal, Université de Montréal, 1000 St-Denis, Montreal, QC H2X 0C1, Canada; christian.watier@umontreal.ca (C.W.); x.weng@umontreal.ca (X.W.); nazila.bettache@umontreal.ca (N.B.); florence.weber.med@ssss.gouv.qc.ca (F.W.); michele.mahone@umontreal.ca (M.M.); stephanie.forte@umontreal.ca (S.F.); michael.newmarch@umontreal.ca (M.J.N.); 2 Department of Gynecology and Obstetrics, Centre Hospitalier de l’Université de Montréal, Université de Montréal, 1000 St-Denis, Montreal, QC H2X 0C1, Canada; andre.masse@umontreal.ca (A.M.); marie-josee.bedard@umontreal.ca (M.-J.B.); 3 Department of Pediatrics, CHU Sainte-Justine, Université de Montréal, 2900 Boulevard Edouard-Montpetit, Montreal, QC H3T 1J4, Canada; vincent-philippe.lavallee@umontreal.ca; 4 Department of Medicine, Cancer Center of Southeastern Ontario, Queen’s University, 99 University Avenue, Kingston, ON K7L 3N6, Canada; p-ogaudreau@ctg.queensu.ca

**Keywords:** fetal hemoglobin, pregnancy, glycosylated hemoglobin, HbA1C, β-HCG

## Abstract

It is believed that fetal hemoglobin (HbF) expression in adults is largely genetically regulated. The increased expression of HbF in pregnancy has been reported in a small number of articles. Different mechanisms have been proposed, but the description of HbF expression during pregnancy remains unclear. The objectives of this study were to document HbF expression during peri and postpartum periods, confirm its maternal origin, and assess clinical and biochemical parameters potentially associated with HbF modulation. In this observational prospective study, 345 pregnant women were followed. At baseline, 169 had HbF expression (≥1% of total hemoglobin) and 176 did not have HbF expression. Women were followed at the obstetric clinic during their pregnancy. Clinical and biochemical parameters were measured at each visit. Analyses were made to determine which parameters had a significant correlation to HbF expression. Results show that HbF expression of ≥1% during peri and postpartum periods in pregnant women without influencing comorbidities is at its highest peak during the first trimester. In all women, it was proven that HbF was of maternal origin. A significant positive correlation between HbF expression, βeta-human chorionic gonadotropin (β-HCG), and glycosylated hemoglobin (HbA1c) was present. A significant negative association between HbF expression and total hemoglobin was found. HbF expression induction during pregnancy is probably associated with an increase in β-HCG and HbA1C, and a decrease in total hemoglobin, which could temporarily reactivate the fetal erythropoietic system.

## 1. Introduction

In human adults, fetal hemoglobin (HbF) normally represents approximately 1% of total hemoglobin. The switch from fetal hemoglobin forms (α_2_^G^γ_2_ and α_2_^A^γ_2_) to adult HbA and minor HbA_2_ forms happens in utero in the last weeks of pregnancy. The precise mechanism of this switch is still misunderstood. One proposed mechanism relates to a change in the gene expression of a single population of stem cells [1]. Another implies silencing of the γ-globin gene in reticulocytes. Orneal et al. suggested that the gradual decrease in the γ-globin gene during the fetal period seems to be independent of this silencing [2]. However, there is a temporal relationship between birth and the silencing of the γ-globin gene. Indeed, there is an abrupt postnatal increase in the amount of gene-silenced γ-globin reticulocytes which is presumed to be triggered by an event related to birth, possibly normoxic respiration.

The percentage of HbF in adults can be higher in some hematologic pathologies, such as β-thalassemia, sickle cell disease, and aplastic anemia [3]. In these conditions, the persistence of adult erythrocytes producing fetal hemoglobin (F-cells) can have protective properties. It is believed that the HbF and F-cells percentage in adults are substantially genetically regulated [1]. However, HbF synthesis regulation mechanisms in adults remain misunderstood despite the underlying therapeutic potential for hemoglobinopathies. Pregnancy is the only known non-pathological condition in which HbF level can transiently increase in adults, either by feto-maternal transfusion or by physiologic expression. It has been observed that this increase during pregnancy happens both in normal pregnancies and pregnancies of women with hemoglobinopathies [4,5]. The maternal origin of the HbF at the beginning of pregnancy has been reported in many studies. Several hypotheses have been put forward to explain this expression. Among those, pregnancy expression of HbF has been linked to an increase in variable hormones (human chorionic gonadotropin, medroxyprogesterone acetate, and prolactin), erythropoiesis and expansion of maternal erythrocytes mass, and stress-induced temporary reactivation of fetal erythropoietic system [5,6,7,8,9]. However, some of these observations have been denied by others [10,11]. The number of articles and the associated sample sizes that have reported this phenomenon are quite small. Therefore, the description of HbF expression during pregnancy remains unclear. These premises motivated this study, which was conducted in women followed during pregnancy in order to describe and analyze their level of HbF. The objectives of this study were the following: to document HbF expression during peri and postpartum periods, to confirm its maternal origin, and to assess clinical and biochemical parameters potentially associated with HbF modulation.

## 2. Materials and Methods

### 2.1. Parturients Selection

This study is an observational single-institution prospective study. Inclusion criteria are defined as followed: to be a woman of 18 years old or more, to be pregnant in the first trimester of pregnancy, as shown by a fetal heartbeat on ultrasound or a positive urine dipstick βeta-human chorionic gonadotropin (β-HCG) test, and to be followed at the obstetric clinic of Hôpital St-Luc of the CHUM (Centre Hospitalier de l’Université de Montréal). Exclusion criteria are defined as the presence of an abnormal form of hemoglobin (HbC, HbS, HbH or β-thalassemia minor, major or intermedia) as determined by high-performance liquid chromatography (HPLC), patients with type 1 diabetes, patients with myeloproliferative or myelodysplastic syndromes, use of immune suppressors (tacrolimus, azathioprine, cyclosporine or mercaptopurine), creatinine > 140 μmol/L and hepatic disease. Eight hundred and seventy-nine pregnant women were screened to participate from March 2014 to January 2016. The sampling continued until approximately 150 HbF-positive women were listed. Approximately 150 of the first HbF-negative parturients during the sampling were followed during the whole pregnancy to have a sufficient comparator sample size. Overall, at the first visit, a total of 169 women had HbF expression and 176 women did not have HbF expression. Negative total HbF expression was defined as a <1% HbF level of total hemoglobin and positive total HbF expression was defined as a ≥1% HbF level of total hemoglobin.

### 2.2. Study Procedures

Women were followed during four visits at the obstetric clinic. The three first visits were at each trimester (8–12, 24–28, and 36th gestational weeks) and HbF-positive women had an additional 6 week postpartum visit to assess for hereditary persistence of HbF (HPFH). Consent and information forms were presented and signed by the parturients at the first visit. Clinical characteristics were identified at the first and consecutive visits: age, weight (kg), date of latest menstruation, smoking history, primiparous or multiparous status, presence of multiple pregnancies, hypertension history, and history of a fertility clinic consultation. The level of creatinine was only measured at the first visit. At each following visit, the following data were also noted: gestational week, presence or absence of active hypertension, diabetes, eclampsia or preeclampsia, and use of progesterone. The baby’s weight (kg) was also measured. Gestational age was determined by the date of the latest menstruation and first-trimester fetal ultrasound. Hypertension was defined as a systolic pressure > 140 mm Hg or a diastolic pressure > 90 mm Hg. Preeclampsia was defined as the presence of hypertension with proteinuria of >0.3 g in a 24-h urine collection after 20 weeks of gestation. Eclampsia was defined as generalized seizures or coma in a pregnant woman with diagnosed preeclampsia, in the absence of another cause. Type II diabetes was diagnosed if women had fasting blood glucose > 7 mmol/L or blood glucose > 11 mmol/L after a 2-h glucose challenge test. Gestational diabetes was diagnosed if parturients had at least two of the following criteria: fasting blood glucose of >5.3 mmol/L, blood glucose of >10 mmol/L 1 h after a glucose challenge test, blood glucose of >8.6 mmol/L 2 h after a glucose challenge test or blood glucose of >7.8 mmol/L 3 h after a glucose challenge test. Using specialized questionnaires created for the study, an obstetric nurse at the follow-up clinic collected the data.

### 2.3. Laboratory Evaluations and Measurement of Hemoglobin Components

The following laboratory results were measured at each visit: hemoglobin, hematocrit (Ht), red blood cells level (RBC), red cell distribution width (RDW), mean corpuscular volume (MCV), mean corpuscular hemoglobin concentration (MCHC), mean corpuscular hemoglobin (MCH), reticulocyte count, ferritin, β-HCG level, erythropoietin level (EPO), and glycosylated hemoglobin (HbA1C). At each visit, blood samples were analyzed by HPLC to detect HbF, HbA, HbA_2_, and to quantify HbA1C. The percentage of HbF was measured and defined by negative (<1%) or positive (≥1%) at every visit. The samples that were HbF-positive were analyzed by flux cytometry to determine the origin of the HbF (fetal versus maternal) and to quantify the percentage F-cells. For HPLC, we used the automated analyzer *Bio-Rad Variant II*, with the double program HbA_2_/HbA1C. During the procedure, the time of retention of the hemoglobins as well as the total area of the hemoglobin curves, with target values between 1 and 3.5 million, were monitored. The device pressure was maintained between 35–40 kg/cm and the temperature at 28 °C. The cartridges were calibrated every 250 injections and replaced after every 500 injections. Regarding flux cytometry, we used intracellular labelling with anti-HbF monoclonal antibody (MHFH05, Caltage Laboratories). Initially, 2.5 × 10^7^ peripheral blood cells were fixed with 1 mL of 15% glutaraldehyde solution for 10 min at room air temperature. This preparation was cleaned thrice with a Phosphate Buffered Saline solution with Bovine Serum Albumin (PBS-BSA) 1%. It was then suspended in 0.5 mL of Triton X-100 solution and incubated 3-to-5 min at room temperature. The sample was cleaned once again and suspended in 0.5 mL of PBS-BSA 1%. After, 10 μL of this suspension was added to 5 μL of antibody and 10 μL of PBS-BSA 0.1%. The marked red blood cells were stored in 0.5 mL of 1% formaldehyde, then stored in the dark before being analyzed by the cytometer.

### 2.4. Statistics

Descriptive statistics were used to present the characteristics of HbF-positive and negative women. The comparisons were made at first, second, and third visits, and at any time, assessing the proportions of positive and negative cases. Means were calculated on all demographic values and blood parameters. Multiple analyses of covariance (ANCOVA) were performed between each of the following parameters and demographic and medical data: HbF%, F-cells%, HbA1C, total hemoglobin, EPO and β-hCG levels. Both HbF-positive and negative groups were included in those analyses. For the HbF-positive group, Pearson correlations were made to predict the HbF%, based on F-cells%, HbA1C%, EPO and β-hCG level, total hemoglobin, and multiple blood parameters (reticulocytes, Ht, RBC, MCHC, MCH, RDW, MCV, and ferritin). The bilateral *p*-value significance was set between 0.01 and 0.05, depending on the correlation studied. Student *t*-tests were performed on significant correlations to determine if a significant difference was present between the pregnancy periods (at each of the four visits). Additional *t*-tests were performed on parameters strongly correlated with the expression of HbF, to compare the mean level of the parameter in the HbF-positive and HbF-negative groups. Among the Pearson correlations with significant results, multivariate analyses were made using linear regressions.

## 3. Results

### 3.1. Descriptive Statistics

Among all pregnant women screened (*n* = 879), 22.07% (*n* = 194) had positive HbF expression. Among those HbF-positive women, 18 were non-eligible. Thus, among screened women, 20.02% (*n* = 176) filled the inclusion criteria. The total number of women included in the comparative study was 345: 51.01% (*n* = 176) were HbF-positive women and 48.99% (*n* = 169) were HbF-negative initially. Including the women who became HbF-positive at the second follow-up visit among the women who were initially HbF-negative, 22.53% (*n* = 198) of 879 screened women were HbF-positive. Analyzed at the time of the second visit, of the 345 women included, 57.39% were HbF-positive. Therefore, 13.02% of the 169 initially HbF-negative women became HbF-positive at the second visit. At the time of analysis, 155 HbF-positive and 147 HbF-negative women had completed follow-up for a total of 302 women (Table 1).

The median HbF percentage of HbF-positive women was 1.2% at the first visit, 1.0% at the second visit, 0.8% at the third visit, and 0.7% at the fourth visit. For all visits, the median HbF% of positive women was 1.0% (Table 2). Among the 176 originally HbF-positive women, 155 completed the fourth visit follow-up. At this visit, 113 out of 155 HbF-positive women (72.9%) showed a postpartum decrease in HbF < 1%. The means of demographic values and blood parameters are presented in Table 3 and Table 4.

### 3.2. F-Cells Percentage

F-cells analyses confirmed the maternal origin of HbF in all cases. At the first visit, the mean F-cells percentage in HbF-positive women was 13.89%. Other means were 13.48% at the second visit, 12.22% at the third visit, and 11.48% at the fourth visit. For all visits, the mean F-cells percentage was 13.3% (standard deviation = 5.38%) when total HbF was ≥1% during pregnancy (Table 5).

### 3.3. Pearson Correlations and t-Tests

**Figure 1:**
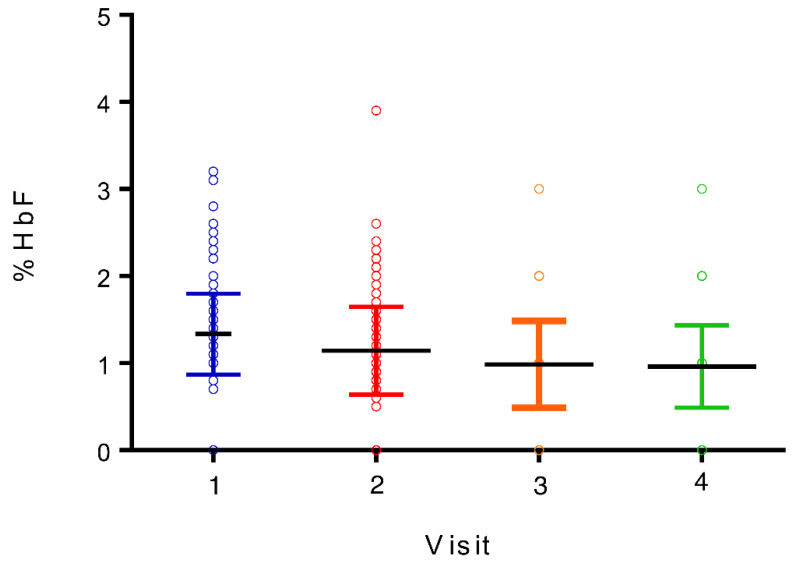
HbF percentage by HPLC for HbF-positive parturients.

**Figure 2:**
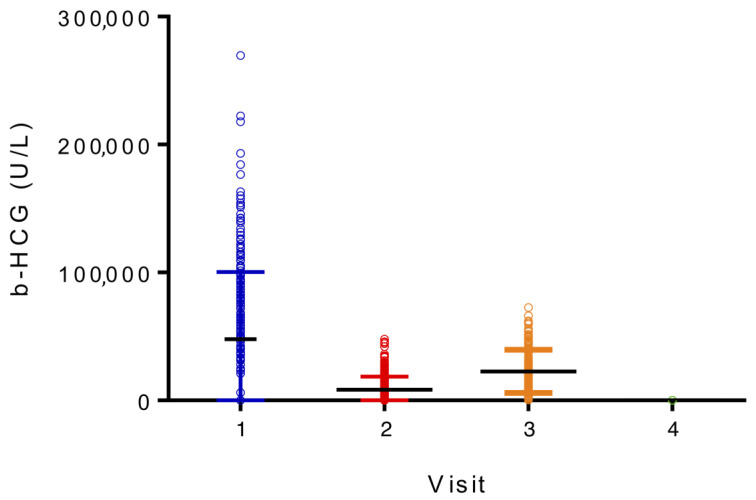
β-HCG per visit for HbF-positive parturients.

Many independent *t*-tests and Pearson correlations showed that the link between multiple demographic data and the HbF% at the first visit was not significant (Table 6). However, for all parturients included, significant variables linked with HbF expression were β-HCG, MCH, RDW, HbA1C, and ferritin. For the HbF-positive group, the results show a significant positive association between HbF expression and β-HCG, HbA1C, MCH, and RDW, as well as a significant negative association between HbF expression and total hemoglobin (Table 7, Figure 1 and Figure 2). Those results had a *p*-value of 0.00001.

## 4. Discussion

The objectives of this study were met to document HbF expression during peri and postpartum periods, to confirm its maternal origin, and to assess clinical and biochemical parameters potentially associated with HbF modulation. To our knowledge, this study depicts the largest cohort description of the expression of HbF in pregnant women. Indeed, the sample size and the number of characteristics studied are sufficient to adequately describe the prevalence and duration of this biological behavior. Moreover, this study also represents the longest longitudinal evaluation of HbF expression during peri and postpartum periods in the literature. It is also the first study reporting the physiologic expression of HbF in pregnancy studied by HPLC. The current study has wide applicability to pregnant women without comorbid conditions as defined in the exclusion criteria.

The results show that at the first-trimester visit, 176/879 (20.02%) screened women were HbF-positive. At any time during the total evaluation, 198/879 (22.53%) screened women were HbF-positive. At the postpartum visit, 72.9% of HbF-positive women showed a decrease in HbF < 1%. This observation allows us to exclude the hereditary persistence of fetal hemoglobin syndrome as the cause of higher HbF percentage in participating parturients. Additionally, flux cytometry confirmed the maternal origin of HbF in all cases as expected because HbF is not expressed at this time in pregnancy in the fetus. For all visits, the mean of erythrocytes expressing fetal hemoglobin was 13.3% (representing the mean F-cells percentage). Given the considerable sample size, these results are likely to represent the reality regarding HbF expression in pregnant women and serve as a reference for the future.

The results evidence that 20.02% of pregnant women express HbF. This proportion is higher than the expected 1/8 based on previous reports in the literature [5,6,8,9]. It is even more striking since the selection criteria excluded women with hemoglobinopathies and type 1 diabetes, known populations that have a higher proportion of women with an HbF percentage of ≥1% [12].

The data also provide evidence that there is a significant positive association between HbF expression in pregnant women and β-HCG, HbA1C, MCH, and RDW. However, there is a significant negative association between HbF expression and total hemoglobin. The association between β-HCG and HbF has already been described in other reviews. Indeed, Popat established a temporal relationship between β-HCG and HbF rise in pregnancies with and without HCG injections [6]. Similarly, Lee showed that the peaks of β-HCG and HbF, between 9 and 12 weeks of gestation, were concordant [11]. The results of this study present the same pattern, with a spike of β-HCG and HbF during the first trimester in HbF-positive women, declining thereafter. However, other studies have not observed this phenomenon. For example, the β-HCG pattern was identical between pregnant women who showed an increase in HbF expression and those who did not in a study by Pembrey and colleagues [10]. Their sample size was only 54 women and HbF measurement was executed with a modified alkaline denaturation method. Noteworthy to be highlighted is the temporality of the HbF rise. Like Pembrey [10] and Kristoffersen [13], the results of this study show that the maximal expression of HbF was during the first trimester. In Popat [6] and Rucknagel’s [9] studies, this peak was rather in the second trimester, and in Boyer’s [14], it was between the 23rd and 31st gestational weeks. In Lee’s study [11], in addition to the first-trimester major HbF peak, two minor peaks (between weeks 16–20 and 22–24) were demonstrated. Therefore, the timing of the maximal HbF spike seems inconsistent. One reason that could explain this variation is the differing inclusion and exclusion criteria between the studies and the distinct HbF measurement methods used. The results suggest that β-HCG may induce HbF synthesis during pregnancy, but the precise mechanism remains unknown. A bystander effect is possible, but a hormone-responsive rise in HbF is certainly a preferred possible mechanism in pregnancy.

The negative relationship between total hemoglobin and HbF expression could be related to the suggested mechanism by Rucknagel and Chernoff of fetal erythropoietic reactivation during pregnancy [9]. They linked this reactivation with the various stressors associated with the beginning of pregnancy, for example, the abrupt rise in β-HCG. A hypothesis for the subsequent gradual decline after the early rise in HbF during pregnancy was the accustoming of the woman’s body to these changes. Various studies have proved the reactivation of HbF synthesis in situations of abrupt expansion of erythropoiesis, such as iron deficiency anemia, hemolytic anemia, and acute bleeding [15,16]. All of these situations correspond to an inverse relationship between HbF expression and total hemoglobin. In an acute phase of erythrocyte repopulation secondary to anemia, it is proposed that the more immature erythrocyte precursors, the Erythroid Burst-Forming Unit (BFU), are temporarily mobilized to enter directly into terminal maturation until the size of the Erythroid Colony-Forming Unit (CFU), the more mature precursors, increase. It was demonstrated in vitro that the presence of BFU is associated with a transient increase in HbF [17,18]. It is thought that the difference in HbF production between BFU and CFU is due to a difference in HbF produced by the F-cells [19]. Anemia is a frequent phenomenon in pregnancy; it is thus possible that the increase in HbF expression in pregnant women is caused by alike mechanisms. However, anemia is usually more pronounced during the third trimester in which expression of HbF is usually not as marked in the results presented here.

A significant positive association between HbF expression and HbA1C was also found in the results. The link between insulin via insulin growth factor 1 (IGF-1) and erythropoiesis has already been well established by previous studies, both in vitro and in vivo [20]. It is believed that IGF-1 acts as an erythropoiesis-inducing hormone via EPO dependant and independent pathways, as shown in in vitro human erythroid colony formation. This hypothesis is supported by the demonstration of erythropoiesis induction by IGF-1 both in the presence and absence of EPO [21,22]. Regarding in vitro human studies, Miyagawa and colleagues observed that IGF-1 stimulated erythropoiesis from the terminal stage of BFU to the stage of CFU [23]. They suggested that insulin and IGF-1 could act as a burst-promoting activity on BFU. In parallel, various authors have already shown the correlation between higher HbF percentage and diabetes, particularly in type 1 diabetes. Koskinen measured that in a population of 1104 patients with type 1 or 2 diabetes, among adults, 6.5% of them had an HbF > 1% [13]. Some characteristics, such as younger age, type 1 diabetes, and treatment with insulin, seem to be more strongly associated with HbF expression. Pardini and Wise also established the association between insulin and a higher HbF expression [24,25]. Perrine and colleagues suggested that insulin as well as other growth factors such as EPO could cause a change in the production sequence of β-globin gene chains. The finding that the production of β-chains is delayed in newborns of diabetic mothers supports this hypothesis. Indeed, it suggests the persistence of γ-chain production synthesis in the context of hyperinsulinism [26]. Interestingly enough, for a long time, the only non-experimental treatment used for gestational diabetes besides lifestyle changes was insulin. The relation between gestational diabetes and HbF expression has been less reported than the one with type 1 and 2 diabetes. The study shows that there is a significant relation between HbF expression and HbA1C in pregnant women, which could be linked to the regulatory effect of insulin and IGF-1 on human erythropoiesis, as described before. However, to our knowledge, there has not been any research that studied the specific mechanism of IGF-1 during pregnancy yet.

This study is associated with some limitations, but the difference in results compared with previous studies is balanced by the large sample size of the current demonstration. However, one source of variability compared to previous reports concerns the use of HPLC. One study has shown that HbF levels determined by HPLC were inaccurate in the presence of increased HbA1C in 41% of diabetic patients tested [27]. The risk of false positives was greater if the HbA1C% was higher, especially in severe cases with HbA1C measuring between 11.1 and 19.8%. The authors found that this false rise could be predicted by an HbF retention time of over 1.15 min. However, this source of error should not have greatly influenced this study’s results since the HbA1C% of the parturients was between 3.7 and 6.4%.

The cause of HbF synthesis induction during pregnancy probably lies within a mix of different factors. β-HCG, acute anemia, and elevated HbA1C are probably some of those associated with the modulation of fetal hemoglobin synthesis. This points to polymorphism in genes associated with HbF production, either directly or indirectly. These could be hormone receptors as well as proteins affected by anemia. Future genetic analyses with the sequencing of multiple genes of different HbF loci will help to elucidate these mechanisms.

## 5. Conclusions

In summary, this study showed that HbF expression of ≥1% during peri and postpartum periods in pregnant women without influencing comorbidities is at its highest peak during the first trimester. At any time in the study, the highest percentage of HbF-positive (≥1%) women among included parturients was 22.53%. The HbF percentage decreased below 1% in 72.9% of positive women in the postpartum period, therefore excluding the hereditary persistence of fetal hemoglobin. In all women, it was proven that HbF was of maternal origin. The results show a significant positive correlation between HbF expression and β-HCG as well as HbA1C, and a significant negative association with total hemoglobin. Fetal hemoglobin expression induction during pregnancy is probably associated with these elements, which could temporarily reactivate the fetal erythropoietic system. To our knowledge, this study has the largest sample size and longitudinal evaluation that has ever been reported on fetal hemoglobin expression during pregnancy as of today. Therefore, it provides important reference data. Further investigations are warranted to propose mechanisms leading to the physiologic expression of fetal hemoglobin during pregnancy. Mild fetal hemoglobin induction is common in normal pregnancies and should not be a reason for concern. However, it may be associated with anemia and an increased risk of diabetes. Since these conditions are already systematically screened for during peripartum care, we do not suggest modifying the management of pregnant women with mild HbF induction at this moment.

## Figures and Tables

**Table 1:** Demographic values.

Demographic Value	Detail	Number (%) SD
Age	Mean	32.29 (NA) 4.11
	Minimum	20 (NA)
	Maximum	44 (NA)
	More than 35 years old	255/345 (73.91)
	Less than 35 years old	90/345 (26.09)
History of smoking	Number of parturients	124/345 (35.94)
	Mean of years of smoking in parturients with smoking history	3.28 (NA) 5.62
Mean of number of pregnancies		2.00 (NA) 1.20
Mean of parity		0.57 (NA) 0.72
Multiple pregnancy		2/345 (0.58)
History of anterior fertility clinic consultation		41/345 (11.88)
History of hypertension		10/345 (2.90)
Parturients with hypertension at first visit		3/345 (0.87)
Parturients with active preeclampsia at first visit		0/345 (0)
Parturients with gestational diabetes at first visit		1/345 (0.29)
History of type 2 diabetes		0/345 (0)
Use of progesterone before visit 1		23/345 (6.67)

NA: non applicable; SD: standard deviation.

**Table 2:** Descriptive statistics of the study.

Value	*n*
Number of women screened	879
Number of women included in the study	345
Number of HbF-negative women at first visit selected for comparator group	169
Number of HbF-negative women who completed follow-up at time of analysis	147
Number of HbF-positive women at first visit	176
Number of HbF-positive women at anytime during evaluation	198
Number of HbF-positive women who completed follow-up at time of analysis	155

**Table 3:** Median HbF percentage in HbF-positive women.

Visit	Median Hb F Percentage (%)	Range (%)
1 (weeks 8–12)	1.2	0–3.2
2 (weeks 24–28)	1.0	0–3.9
3 (week 36)	0.8	0–3.2
4 (6 weeks postpartum)	0.7	0–2.9

**Table 4:** Means of blood parameters.

Value	Visit 1 Mean (SD)	Visit 2 Mean (SD)	Visit 3 Mean (SD)	Visit 4 Mean (SD)
Hemoglobin (g/L)	126.2 (8.3)	117.4 (8.0)	120.2 (10.1)	132.2 (8.0)
Hematocrit (L/L)	0.367 (0.022)	0.349 (0.022)	0.356 (0.026)	0.397 (0.022)
RBC (×10^12^/L)	4.15 (0.30)	3.82 (0.28)	3.95 (0.30)	4.43 (0.30)
MCV (fL)	88.8 (3.4)	91.7 (3.7)	90.5 (4.4)	89.9 (3.9)
RDW (%)	12.9 (0.7)	13.3 (0.6)	13.5 (0.8)	13.2 (1.6)
MCHC (g/L)	343.6 (7.9)	336.4 (7.7)	337.4 (9.2)	332.7 (9.2)
MCH (pg)	30.5 (1.3)	30.8 (1.5)	30.5 (1.9)	29.9 (1.6)
Reticulocytes (×10^9^/L)	70.8 (20.7)	82.9 (20.7)	88.5 (19.0)	55.5 (17.3)
Ferritin (ng/mL)	72.2 (48.9)	29.9 (81.2)	20.7 (14.8)	66.8 (47.5)
β -HCG (mUI/mL)	86,344 (41,388)	15,151 (10,549)	22,916 (17,840)	225 (2831)
EPO (UI/L)	9.1 (3.5)	18.9 (11.4)	26.6 (31.1)	7.8 (3.9)
HbA1C (%)	5.095 (2.99)	4.911 (3.482)	5.192 (3.934)	5.318 (3.809)

RBC: red blood cells count/MCV: mean corpuscular volume/RDW: red cell distribution width/MCHC: mean corpuscular hemoglobin concentration/MCH: mean corpuscular hemoglobin/β-HCG: beta human chorionic gonadotropin/EPO: Erythropoietin/; HbA1C: glycosylated hemoglobin/SD: standard deviation.

**Table 5:** F-cells percentage in HbF-positive women.

Visit	Minimum (%)	Maximum (%)	Range (%)	Mean (%)
1 (weeks 8–12)	4.0	32.8	28.8	13.89
2 (weeks 24–28)	4.0	30.2	26.2	13.48
3 (week 36)	5.2	26.9	21.7	12.22
4 (6 weeks postpartum)	4.8	22.8	18.0	11.48
All visits	4.0	32.8	28.8	13.30

**Table 6:** Independent *t*-tests and Pearson correlations between demographic data and HbF% at the first visit.

*t*-Tests Relation	*t* Value	*p*-Value
HbF-Positive/Negative women and total hemoglobin	NA	NS
Age (above or below 35 years-old) and HbF%	−0.114144	0.909190
Smoking status and HbF%	1.787341	0.074770
Fertility clinic consultation and HbF%	1.189824	0.234939
Hypertension history and HbF%	−0.451676	0.651788
Use of progesterone and HbF%	0.916958	0.359809
**Pearson Correlations Relation**		***p*** **-Value**
Weight and HbF%		0.05
Number of years of smoking and HbF%		0.05
Number of pregnancies and HbF%		0.05

NS: non-significant; NA: non applicable.

**Table 7:** Correlation coefficients of multivariate analyses between HbF, F-Cells percentage, and various variables.

Ascending Stepwise Regression Variables (All Visits)	*p*-Value	Adjusted Correlation Coefficient (R^2^)
All parturients and all variables to predict HbF%	0.0041	62.20%
All parturients and significant variables to predict HbF%	0.0010	58.44%
All parturients and all variables to predict F-cells%	0.0539	76.49%
All parturients and significant variables to predict F-cells%	0.1139	68.29%
All parturients and all variables to predict F-cells intensity	0.0141	64.99%
All parturients and significant variables to predict F-cells intensity	0.0141	64.99%
HbF-positive parturients and all variables to predict HbF	0.0013	16.14%
HbF-positive parturients and significant variables to predict HbF	0.0000	15.18%
HbF-positive parturients and all variables to predict F-cells%	0.0567	76.53%
HbF-positive parturients and significant variables to predict F-cells%	0.9977	75.87%
HbF-positive parturients and all variables to predict F-cells intensity	0.0140	64.98%
HbF-positive parturients and significant variables to predict F-cells intensity	0.0140	64.98%

All variables: β-HCG, MCV, RBC, HbA1C, EPO, MCHC, Hb, Ferritin, EPO; Significant variables: β-HCG, MCV, HbA1C, MCHC, Hb.

## Data Availability

The data presented in this study are available in Prospective Evaluation of Fetal Hemoglobin Expression in Maternal Erythrocytes: an Analysis of a Cohort of 345 Parturients, Blain Laurence, Soulières Denis and Al.
